# Clinical Effectiveness and Safety of Chinese Herbal Medicine Compound Kushen Injection as an Add-On Treatment for Breast Cancer: A Systematic Review and Meta-Analysis

**DOI:** 10.1155/2022/8118408

**Published:** 2022-01-10

**Authors:** Bao-Yong Lai, Ai-Jing Chu, Bo-Wen Yu, Li-Yan Jia, Ying-Yi Fan, Jian-Ping Liu, Xiao-Hua Pei

**Affiliations:** ^1^Third Affiliated Hospital of Beijing University of Chinese Medicine, Beijing 100029, China; ^2^The Xiamen Hospital of Beijing Universality of Chinese Medicine, Xiamen 361001, China; ^3^First Affiliated Hospital of Xiamen University, Xiamen 361003, China; ^4^Centre for Evidence-Based Chinese Medicine, Beijing University of Chinese Medicine, Beijing 100029, China

## Abstract

**Objective:**

To systematically evaluate the effect and safety of compound Kushen injection (CKI) as an add-on treatment on the treatment for breast cancer.

**Methods:**

We searched eight major electronic databases from their inception to November 1, 2021, for randomized clinical trials (RCTs) comparing CKI plus chemotherapy with chemotherapy alone. Primary outcomes included objective response rate (ORR) and disease control rate (DCR), health-related quality of life (HRQoL), progression-free survival (PFS), and overall survival (OS). Secondary outcomes included adverse drug reactions (ADRs) and tumor marker level. We used Cochrane's RevMan 5.3 for data analysis. The GRADEpro was used to appraise the certainty of evidence. Trial sequential analysis (TSA) was applied to estimate the required sample size in a meta-analysis and test the robustness of the current results.

**Results:**

Thirty RCTs with 2556 participants were totally included. CKI plus chemotherapy showed significant effects in increasing ORR (RR 1.30, 95%CI [1.18, 1.43], *I*^*2*^ = 27%, *n* = 1694), increasing DCR (RR 1.21, 95%CI [1.15, 1.28], *I*^*2*^ = 16%, *n* = 1627), increasing HRQol as measured by Karnofsky Performance Scale (KPS) score improvement rate (RR 1.42, 95% CI [1.26, 1.61], *I*^*2*^ = 37%, *n* = 1172), increasing the PFS (MD 2.24 months, 95%CI [1.26, 3.22], *n* = 94) and the OS (MD 2.24 months, 95%CI [1.45, 3.43], *n* = 94), compared to chemotherapy alone. The results showed that CKI plus chemotherapy had a lower risk of ADRs than that of chemotherapy alone group. The certainty of evidence of the included trials was generally low to very low. TSA for ORR and KPS score improvement rate demonstrated that the current results reached a sufficient power regarding both numbers of trials and participants.

**Conclusions:**

Low certainty of evidence suggested that the combination of CKI and conventional chemotherapy appeared to improve ORR, DCR, and KPS score in breast cancer patients. Conclusions about PFS and OS could not be drawn due to lack of evidence. Additionally, CKI appeared to relieve the risk of ADRs in patients with breast cancer receiving chemotherapies. However, due to weak evidence, the findings should be further confirmed in large and rigorous trials.

## 1. Introduction

Breast cancer is one of the most common cancers experienced by women and is reported as the leading cause of cancer-related death in women around the world [1, 2]. The incidence rate increased significantly in the last decade, with more than 1 million women newly diagnosed with breast cancer every year [3]. Therefore, breast cancer has been a serious burden for societies in the world. Current treatments for breast cancer mainly included surgery, chemotherapy, radiotherapy, hormone, and immunological therapy [4]. However, it was reported that these treatments usually bring about significant side effects, including cardiac toxicity, gastrointestinal toxicity, and other multiple adverse drug reactions [5–7]. All these seriously affected the patients' quality of life, work, and other health outcomes. Therefore, finding alternative options to alleviate the side effects of conventional therapies and improve the clinical efficacy are needed and expected.

Traditional Chinese medicine (TCM), as an add-on treatment, has been increasingly used for the treatment of tumors [8, 9]. Among TCM treatments, compound Kushen injection (CKI) (drug approval number: Z14021231, China food and Drug Administration), an important Chinese herb injection, is used extensively for multiple malignant tumors in China nowadays. Especially in the treatment of breast cancer, it has become increasingly popular TCM injection [10, 11]. CKI is composed of the extracts from *Kushen* (Radix Sophorae Flavescentis) and Baituling (Rhizoma Smilacis Glabrae). Their main active ingredients are matrine, oxymatrine, sophocarpine, and sophoridine, which have been reported to exhibit a variety of pharmacological activities such as a good synergistic antitumor eﬀect [11, 12]. Furthermore, previous studies have revealed that CKI can inhibit the proliferation, invasion, and migration of breast cancer cells through different mechanisms [12, 13].

Some clinical trials have been conducted to investigate the effect of CKI on treating breast cancer. The systematic reviews in 2018 and 2019 [14, 15] involving 24 trials and 18 trials, respectively, showed that CKI had a beneficial effect on improving the working status of patients with breast cancer, and the combination therapy also had a lower risk of adverse drug reactions (ADR) (such as neutropenia, thrombocytopenia, and nausea/vomiting). However, some nonrandomized controlled trials were mistakenly included for analysis, and the results demonstrated that the available data provided insufficient evidence to support the use of CKI for the treatment of breast cancer. On the other hand, the studies included in the systematic review did not report on important outcomes such as the disease control rate (DCR) and long-term survival, which increased the limitation of interpretation and application of the findings. Another recent systematic review [16] involving 16 trials published in 2019 summarized evidence of CKI for breast cancer. The pooled results indicated that the combination of CKI and chemotherapy might improve performance status and reduce ADRs among postoperative patients with breast cancer, but might not improve clinical response rate. In addition, the review finally included trials from 2009 to 2017 and has not been updated since relevant databases were searched from their inception to 2017. Since then, there have been many studies on the effect of CKI on breast cancer. Therefore, this review aims to systematically collect all relevant RCTs to further confirm whether CKI combined with chemotherapy can improve the tumor responses and survivals and reveal its safety.

## 2. Methods

The protocol of this study has been registered at PROSPERO (NO: CRD42020216652). This systematic review was reported in accordance with PRISMA guidelines [17], and we followed the methods of Cochrane methodological guidelines and a previous study published by B.Y. Lai et al. [18, 19].

### 2.1. Inclusion Criteria

#### 2.1.1. Type of Study

This review included randomized clinical trials (RCTs) enrolling participants with breast cancer.

#### 2.1.2. Type of Participants

Type of participants included women who were diagnosed with breast cancer by histopathological and cytological diagnostic criteria, regardless of their age, race, and disease TNM stage.

#### 2.1.3. Type of Interventions

The experimental groups were treated with CKI plus chemotherapy, and the control groups were treated with chemotherapy alone.

#### 2.1.4. Type of Outcomes

Primary outcomes were tumor response (including objective response rate (ORR) and disease control rate (DCR)), health-related quality of life (HRQoL) and long-term survival. For the review, tumor response should be measured using standard evaluation criteria (WHO guidelines or RECIST) and reported as either complete response, partial response, or stable disease or progressive disease [20, 21]. ORR includes participants measured by complete response and partial response. DCR includes participants measured by complete response, partial response, and stable disease. HRQoL could be measured by any recognized evaluation tool. We also considered “Karnofsky Performance Scale (KPS) score improvement” defined as KPS score of more than 10 points increase after treatment as an indicator of HRQoL [16, 21]. Long-term survival was measured by overall survival (OS) or progression-free survival (PFS). Secondary outcomes included adverse drug reactions (ADRs) and tumor marker level. ADRs were summarized according to common terminology criteria for adverse events version (CTCAE) (i.e., the hematotoxicity, liver or renal injury, nausea and vomiting, oral mucositis, and alopecia) [22]. The decrease of tumor marker level' was defined as the tumor marker level decreased more than 25%, or the result recovered from abnormal range to normal range.

Studies were excluded if (1) data could not be extracted; (2) studies where the outcome assessment was not clearly stated; and (3) duplicates.

### 2.2. Search Strategy

We systematically searched major electronic databases (including PubMed, Embase Database, Cochrane library, Web of science, Chinese Biomedical Literature Database (SinoMed), China National Knowledge Infrastructure (CNKI), Wanfang Database and China Science Technology Journal Database (VIP)) from their inception to November 1, 2021. Clinical trial registration platforms (including ClinicalTrials.gov (http://www.clinicaltrials.gov) and Chinese Clinical Trial Registry (http://www.chictr.org/cn)) were also searched for potentially eligible studies. The search terms included “matrine injection,” “compound Kushen injection,” “yan shu injection,” “radix sophorae flavescentis injection,” and “breast cancer.” For example, PubMed was searched with the following search strategy: ((((compound kushen injection [Title/Abstract]) OR (matrine injection [Title/Abstract])) OR (yan shu injection [Title/Abstract])) OR (radix sophorae flavescentis injection [Title/Abstract])) AND (breast cancer [Title/Abstract]). English and Chinese language publications were included.

### 2.3. Study Selection and Data Extraction

Two authors independently selected the studies according to the eligibility criteria. We conducted data extraction using a self-developed data extraction form, and the extracted data mainly included first authors and year of publication, sample size, characteristics of participants, information of randomization, details of intervention, and outcome assessments. If the necessary data were not available in the publication papers, further information was obtained by contacting the first or corresponding author. Any disagreement regarding study selection and data extraction was resolved through discussion.

### 2.4. Assessment of Risk of Bias

The risk of bias of eligible trials was assessed according to the criteria from the Cochrane Handbook for Systematic Reviews of Interventions [18]. Criteria included the following seven domains: random sequence generation, allocation concealment, blinding (including blinding of participants, personnel, and outcome assessors), incomplete outcome data, selective reporting, and other bias (e.g., the comparison of the baseline information). The trials were categorized to high risk of bias when at least one of the items being assessed as “high.” A judgment of low risk of bias of trials was made when all the items met the criteria, and the trial was categorized to unclear risk of bias if insufficient information was obtained for assessment. Any disagreements were resolved by discussion. Finally, the certainty of evidence across studies of each important outcome in this review was appraised using the GRADEpro tool (https://gradepro.org/).

### 2.5. Data Analysis

We used RevMan software 5.3 provided by the Cochrane collaboration to perform statistical analyses. The binary outcomes were expressed as risk ratio (RR) with 95% confidence interval (CI), and the continuous outcomes were expressed as mean difference (MD) with 95% CI. We used the *I*^*2*^ test to detect statistical heterogeneity in effect sizes between studies, and an *I*^*2*^ >50% indicated the possibility of statistical heterogeneity among the studies [23]. Meta-analysis was performed only when there was no significant difference between participants and had acceptable statistical heterogeneity (*I*^*2*^≤75%). Considering potential sources of clinical heterogeneity, the random-effect model (REM) was adopted for meta-analysis in this review. When *I*^2^ >75%, meta-analysis would not be conducted, and individual study results were given, respectively [23]. Then subgroup analysis was performed by disease TNM stage or tumor responses criteria if enough randomized trials were identified and data were available. Furthermore, to estimate the required sample size in a meta-analysis and to test the robustness of the current results, the trial sequential analysis (TSA) was performed if there were more than eight studies in a meta-analysis [24].

## 3. Results

### 3.1. Study Selection

**Figure 1:**
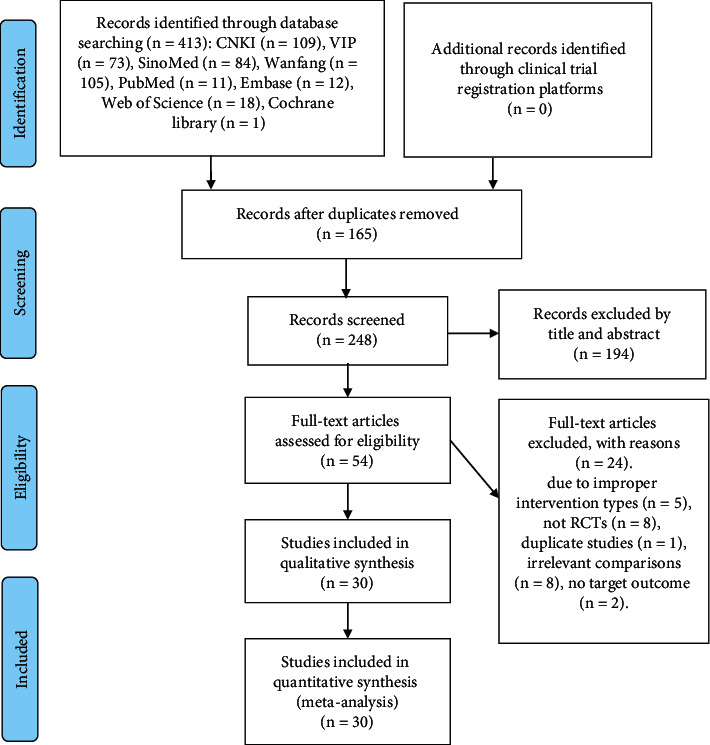
Study selection flow diagram.

A total of 413 studies were identified from eight electronic databases. First, 165 duplicates were excluded, and then 248 were excluded by abstracts reading. Full texts of 54 articles were screened according to the eligibility criteria. Thirty RCTs were included finally. The study searching and selecting process is shown in Figure 1.

### 3.2. Study Characteristics

Thirty RCTs [25–54] involving 2556 participants were included in this review. The sample size varied from 24 to 130 participants, with an average of 85 patients per trial. The mean age of participants was 46.48 ± 6.67 years old based on 14 trials [31, 32, 35, 37, 39, 42, 43, 46, 48–53] reporting age. The dose of CKI was 12–30 ml each time. The treatment time per cycle was from 6 days to 21 days, and the treatment cycle was 2–8 cycles of intravenous injection. None of the trials specified the calculation of sample size. All trials were carried out in China from 2007 to 2020. The characteristics of the included trials are shown in Table 1.

### 3.3. Methodological Quality in Included Trials

**Figure 2:**
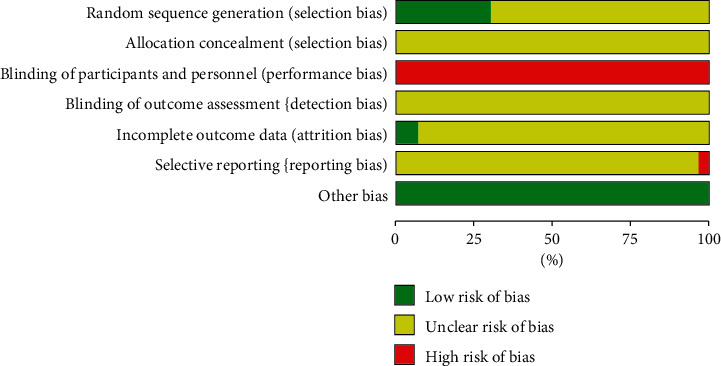
Risk of bias graph.

**Figure 3:**
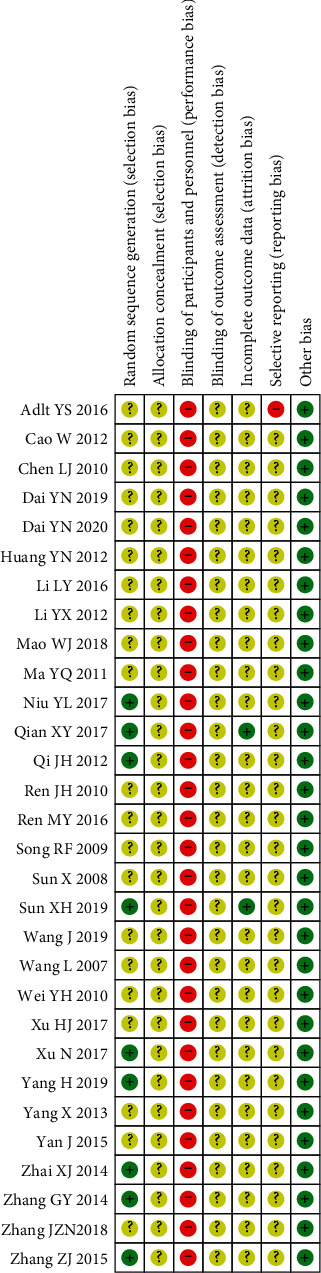
Risk of bias summary.

According to the Cochrane risk of bias tool, the included trials were found to be high risk of bias due to inadequate reporting of the methodological components. Only 9 trials [35–37, 42, 46, 50–53] described that the method of randomization used was assessed as having low risk of bias. It was not possible to blind the participants or personnel as CKI was only used in the experimental group. No trials reported the information of allocation concealment and the blinding of outcome assessment, so the risk of bias on them was judged as “unclear.” Two trials [37, 42] reported participant dropout rate, and the reasons were regarded as low risk of attrition bias. The other trials did not specify the dropout and were all judged as unclear risk of attrition bias. For selective outcome reporting bias, one trial [25] was assessed as high risk of selective reporting bias since it reported primary outcome of long-term survival but failed to report the detailed information. The remaining trials were assessed as having unclear risk of selective reporting bias due to unavailable trial registry. Other bias was assessed by comparability between groups on baseline data, and all trials had baseline comparability. The methodological quality of the included studies is presented in Figures 2 and 3.

### 3.4. Effect Estimates

#### 3.4.1. Primary Outcomes

**Figure 4:**
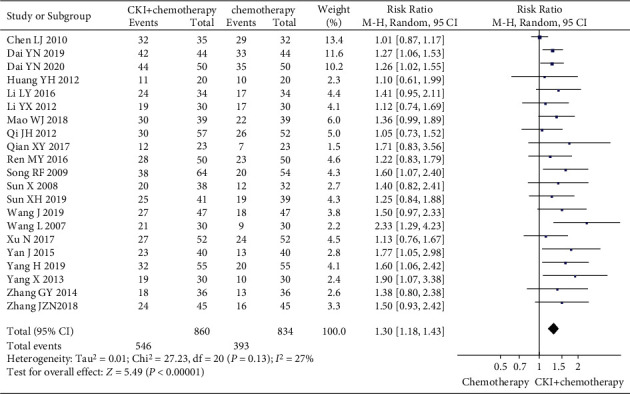
Meta-analysis of ORR of CKI plus chemotherapy for the treatment of breast cancer.

**Figure 5:**
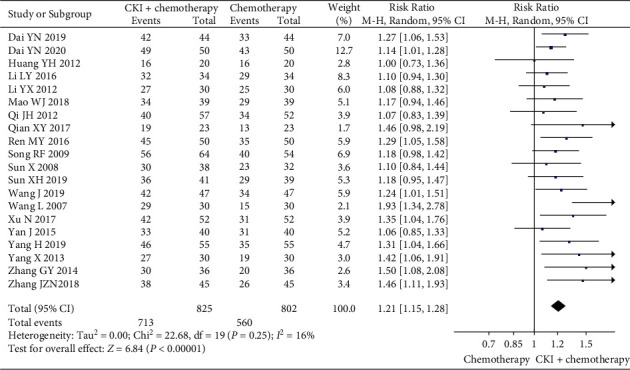
Meta-analysis of DCR of CKI plus chemotherapy for the treatment of breast cancer.

*(1) The Tumor Responses*. The pooled data of 21 trials [27–32, 34, 36, 37, 39–44, 46, 48–50, 53, 54] showed a higher ORR and DCR in CKI plus chemotherapy group than that of chemotherapy alone, and the difference was statistically significant (RR 1.30, 95%CI [1.18, 1.43], *I*^*2*^ = 27%, *n* = 1694, 21 trials, Figure 4) and (RR 1.21, 95%CI [1.12, 1.31], *I*^*2*^ = 71%, *n* = 1694, 21trials). To explore potential causes of the statistical heterogeneity in outcomes of DCR (*I*^*2*^ = 71%), we excluded one trial [27] that reported the same DCR result (100%) in CKI plus chemotherapy group and chemotherapy group, and the remaining trials showed that CKI could increase the DCR (RR 1.21, 95%CI [1.15, 1.28], I^2^ = 16%, *n* = 1627, 20 trials, Figure 5), but the *I*^*2*^ value was reduced to 16%. Thus, we think heterogeneity in this outcome might be caused by participants' baseline conditions or reporting bias.

**Figure 6:**
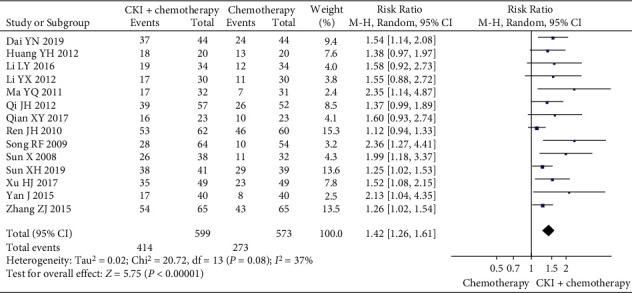
Meta-analysis of KPS score improvement rate of CKI plus chemotherapy for the treatment of breast cancer.

**Figure 7:**
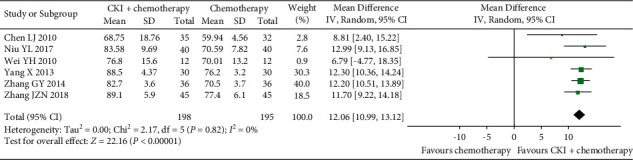
Meta-analysis of KPS score of CKI plus chemotherapy for the treatment of breast cancer.

*(2) HRQoL.* According to the predefined criteria for KPS score improvement, HRQoL was reported as KPS score improvement in 14 trials [28, 30–33, 36–38, 40–42, 47, 48, 52]. A pooled analysis of 14 trials showed a higher KPS score improvement rate in the CKI plus chemotherapy group than that of chemotherapy group alone, and the difference was statistically significant (RR 1.42, 95% CI [1.26, 1.61], *I*^*2*^ = 37%, *n* = 1172, 14 trials, Figure 6). In addition, six trials compared CKI plus chemotherapy with chemotherapy alone [27, 35, 45, 49, 53, 54], and the combination therapy group showed the better effect in improving HRQoL as measured by the KPS score (MD 12.06 score, 95%CI [10.99, 13.12], *I*^*2*^ = 0%, *n* = 393, 6 trials, Figure 7). Similarly, the result from another one trial [43] also showed that CKI plus chemotherapy was superior in improving HRQoL as measured by the QoL-BREF scale (MD 18.11 score, 95%CI [16.50, 18 270 19.72], *n* = 94, Table 2). No other measure of HRQoL was reported in any of the included trials.

*(3) Long-Term Survival.* One trial [43] showed CKI plus chemotherapy was superior in increasing the PFS (MD 2.24 months, 95%CI [1.26, 3.22], *n* = 94, Table 2). Similarly, the result also showed CKI plus chemotherapy was superior in increasing the OS (MD 2.24 months, 95%CI [1.45, 3.43], *n* = 94, Table 2). The findings of another trial [25] were not finally summarized due to this trial reporting no detailed data on the PFS or the OS.

#### 3.4.2. Secondary Outcomes

*(1) Adverse Drug Reactions*. The meta-analysis results showed that CKI plus chemotherapy group had a lower risk of leukocyte decrease (RR 0.60, 95%CI [0.5, 0.71], *n* = 1121, 15 trials), platelet decrease (RR 0.41, 95%CI [0.29, 0.58], *n* = 750, 9 trials), liver injury (RR 0.42, 95%CI [0.31, 0.57], *n* = 1215, 13 trials), renal injury (RR 0.59, 95%CI [0.43, 0.82], *n* = 879, 9 trials), nausea and vomiting (RR 0.68, 95%CI [0.59, 0.79], *n* = 1171, 14 trials), diarrhea (RR 0.55, 95%CI [0.35, 0.88], *n* = 210, 3 trials), alopecia (RR 0.51 [0.39, 0.67], *n* = 584, 8 trials), and oral mucositis (RR 0.18, 95%CI [0.07, 0.45], *n* = 441, 4 trials) than that of chemotherapy alone group. All differences were statistically significant, and the detailed results are shown in Table 2.

*(2) Tumor Marker Level*. The pooled result of three trials [25, 29, 52] showed a higher CEA decrease rate in the CKI plus chemotherapy group than that of the chemotherapy alone group (RR 1.19, 95%CI [1.05, 1.35], *n* = 336, 3 trials, Table 2). Additionally, the pooled data of two trials [25, 28] showed a higher CA15-3 decrease rate in CKI plus chemotherapy group than that of the chemotherapy group (RR 1.15, 95% CI [1.03, 1.27], 2 trials, Table 2).

#### 3.4.3. Subgroup Analyses

Subgroup analyses were performed according to breast cancer TNM stage to reveal the sources of clinical heterogeneity and their influences on ORR, DCR, and KPS score improvement rate. Subgroup analysis indicated a higher ORR and DCR (TNM stage II∼III) in the CKI plus chemotherapy group than that of the chemotherapy alone group from eight trials (Table 2). Similarly, the subgroup analysis also showed a higher ORR and DCR (TNM stage IV) in the CKI plus chemotherapy group than that of the chemotherapy alone group (Table 2).

In addition, subgroup analysis demonstrated that CKI plus chemotherapy group was superior to the chemotherapy alone group in increasing KPS score improvement rate based on TNM stage II∼III or TNM stage IV (Table 2). Finally, tumor responses were evaluated by using WHO criteria or RECIST criteria. The subgroup analysis showed that CKI plus chemotherapy could both increase ORR and DCR according to the two criteria (Table 2).

#### 3.4.4. Certainty of Evidence

We graded the overall certainty of evidence by the GRADE approach for each important outcome. In the comparison of all outcomes and interventions assessments, the certainty of evidence for all outcomes was downgraded to “low” or “very low” mainly due to the high risk of performance bias, the design of comparison and unclear risk of bias for not reporting blinding the outcome assessor. The details of results are shown in Table 3.

#### 3.4.5. Trial Sequential Analysis

**Figure 8:**
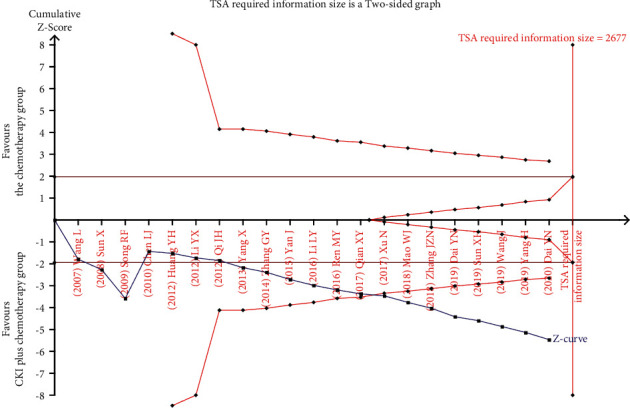
TSA on CKI plus chemotherapy versus chemotherapy for ORR in patients with breast cancer.

**Figure 9:**
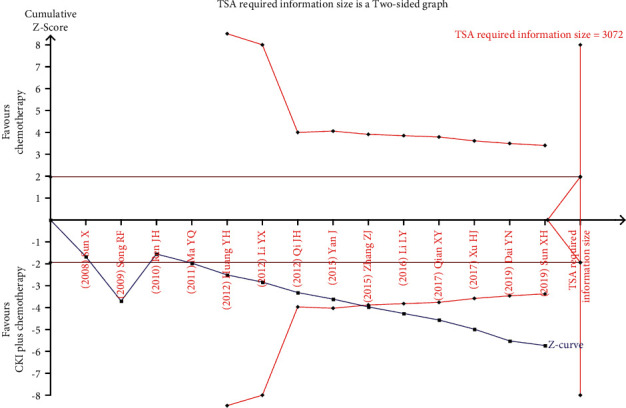
TSA on CKI plus chemotherapy versus chemotherapy for KPS score improvement rate in patients with breast cancer.

TSA was performed with the data from trials reporting the ORR [27–32, 34, 36, 37, 39–44, 46, 48–50, 53, 54]. A required information size was estimated based on Daris type I error = 5%, power = 80%, RRR = 15%, and a two-side graph [24, 51]. The result of TSA illustrated the cumulative Z-curve across the traditional boundary of 5% significance (horizontal red line) and also cross the monitoring boundaries (red inward sloping curves). These indicated that CKI plus chemotherapy for patients with breast cancer could draw an encouraging conclusion on ORR before acquired information size of 2677 participants (Figure 8). Similarly, the result from trials reporting the KPS score improvement rate [28, 30–33, 36–38, 40–42, 47, 48, 52] illustrated that the cumulative Z-curve across the traditional boundary of 5% significance and cross the monitoring boundaries as well, indicating that CKI plus chemotherapy for patients with breast cancer could draw an encouraging conclusion on KPS score improvement rate before acquired information size of 3072 participants [55] (Figure 9).

#### 3.4.6. Publication Bias

**Figure 10:**
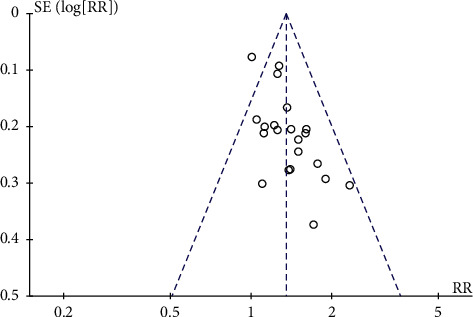
Funnel plot assessing publication bias (using ORR in 21 trials).

**Figure 11:**
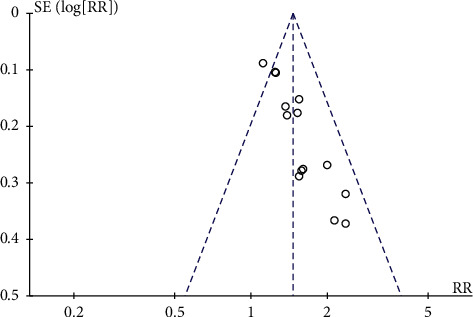
Funnel plot assessing publication bias (using KPS score improvement rate in 14 trials).

The funnel plot was performed using RR and 1/(standard error: SE) values obtained from the trials measuring ORR and KPS score improvement rate. The funnel plots based on ORR and KPS score improvement rate appeared asymmetrical, suggesting the potential publication bias [56] (Figures 10 and 11).

## 4. Discussion

### 4.1. Summary of the Main Results

Thirty trials involving 2556 participants were included in this review. Our results suggested that the combination of CKI and chemotherapy seems to demonstrate the beneficial effect of CKI as an add-on therapy for breast cancer. The tumor response rate (ORR and DCR) and HRQol measured by the KPS score improvement rate in CKI plus chemotherapy group were approximately 14% and 20% higher than that of the chemotherapy group, respectively. The KPS score improvement in CKI plus chemotherapy group was about 12 scores higher than that of the chemotherapy alone group. Long-term effectiveness of the combination of CKI and chemotherapy was insufficiently reported in terms of PFS or OS. CKI plus chemotherapy seemed to be more effective than chemotherapy alone in treating breast cancer. However, the certainty of evidence for all outcomes was mainly assessed as “low” or “very low” due to the risk of bias and statistical heterogeneity among the included studies. The results showed that CKI plus chemotherapy group had a lower risk of ADRs compared to the chemotherapy alone group, and CKI appeared to ease the toxic reaction induced by chemotherapy. However, we could not draw a powerful conclusion from the current evidence.

### 4.2. Strengths and Limitations

Three previous studies [14–16] have assessed the effectiveness of CKI for breast cancer, which demonstrated that CKI used in combined with conventional treatment of chemotherapy seemed more effective than conventional chemotherapy alone. The included trials of the three reviews applied CKI as add-on intervention, and the performance status and tumor response improvement were analyzed. Our findings supported the claims of previous reviews. In contrast, this updated review expanded the trials and sample size, which increased the reliability of synthesized results. Additionally, this review conducted a more rigorous inclusion criterion for participant and outcome assessment and covered additional outcome assessments (such as HRQol, PFS, and OS). Furthermore, we performed subgroup analysis based on the TNM stage and tumor response criteria to reveal the sources of clinical heterogeneity and their influences on primary outcomes such as ORR, DCR, and KPS score improvement rate. This review provided the latest evidence of CKI for treating breast cancer.

There are some limitations of this review. Firstly, all included trials had high risk of performance bias, most of which had unclear risk of selection and attrition bias, which contributed to compromising the trustworthiness and strength of the evidence. Thus, the findings of this review should be interpreted cautiously. Secondly, there were some differences among the dosage of CKI application and chemotherapy regimens in the included trials, which contributed to the increased clinical heterogeneity of the pooled results. Thirdly, our analysis failed to draw conclusions on the long-term survival (PFS or OS) of CKI for breast cancer because there was no evidence to support the effect of CKI for this important clinical outcome in patients with breast cancer. This result needs to be further investigated by new evidence.

### 4.3. Implications for the Clinical Practice

Although there are some potential biases and limitations in this review, the results suggested that CKI as an adjunctive treatment had potential effect in the treatment of breast cancer. According to the results, with the help of CKI, the number of patients with improved tumor response per thousand patients would be almost 140 more than those with the chemotherapy alone. Similarly, the number of patients with improved KPS score per thousand patients in the combination of CKI group would be 200 more than those with the chemotherapy group. Subgroup analysis did not find significant different results between stage II-III and Stage IV for all the concerned outcomes. According to this review, the common dosage of CKI in the treatment of breast cancer was 12–30 ml/time injected intravenously (the general dosage is 20 ml/time). The treatment durations per cycle were 6, 7, 10, 12, 14, 15, or 21 days, and treatment cycles varied from two to eight cycles. Overall, though the certainty of the evidence is “low” or “very low,” the combination of CKI and chemotherapy might be a choice in clinical practice for women with breast cancer because its estimate effect was significantly better than that of the chemotherapy alone. Considering the weak evidence of this intervention, practitioners may consider its use based on their clinical experience and the actual condition of patients.

### 4.4. Implications for Research

The methodological information was reported insufficiently in the included trials. Future trials are encouraged to design, conduct, and report according to the Consolidated Standards of Reporting Trials (CONSORT) statement [57], which is critical to control the risk of bias and improve the reliability of the evidence of CKI in the treatment of breast cancer. Additionally, study protocol should be prospectively registered on authoritative registration platforms before study implementation to ensure the research can be conducted according to the predefined standard [58]. Furthermore, in order to observe the long-term efficacy of CKI in the treatment of breast cancer and evaluate the survival time or progression time of patients, sufficient follow-up time should be considered and reported during the study period. Most importantly, to give conclusive evidence and influence clinical practice, we also suggest future studies report detailed information on PFS, OS, and ADRs, as well as tumor marker level, which are important data for further evaluation of the effectiveness of CKI.

Besides the effectiveness, safety issues related to CKI should not be overlooked. The results of this review indicated that CKI might have a protective effect on liver and kidney function. Based on the quality of current evidence and the related evidence, we believe that CKI could relieve the hematotoxicity and gastrointestinal reactions [10, 59, 60]. However, only about 53% of the included trial reported ADRs during treatment; thus, no firm conclusion on the safety of CKI could be drawn from this review.

## 5. Conclusions

Low certainty of evidence suggested potential effectiveness of the combination of CKI and chemotherapy regimens for treating breast cancer, especially on improving ORR, DCR, and KPS score in patients with breast cancer. The conclusions about PFS or OS could not be drawn according to this review due to lack of evidence. CKI appeared to relieve the risk of ADRs in patients with breast cancer receiving chemotherapies. However, due to weak evidence, the findings should be further confirmed in large and rigorous trials.

## Figures and Tables

**Table 1:** Characteristics of included RCTs on compound Kushen injection for breast cancer.

Study ID	Sample (T/C)	Age (y)	Course of disease (months)	Stage	Control/chemotherapies	Intervention (CKI)	Duration	Tumor responses criteria	Outcome measures
Adlt YS 2016 [25]	T:56	T:34–68	NR	I∼II	AC1	CT + CKI 20 mL, qd,21d	6 cycles	Unclear	PFS, OS, CEA/CA153 level
	C:62	C:30–65							

Cao W 2012 [26]	T:52	T:42–70	NR	II∼III	C1AF	CT + CKI 20 mL, qd,10d	6 cycles	Unclear	ADRs
	C:52	C:45–68							

Chen LJ 2010 [27]	T:35	T: 39–71	NR	II∼III	TC1A	CT + CKI 20 mL, qd,14d	6 cycles	WHO	ORR, DCR, HRQoL-KPS, ADRs
	C:32	C: 35–68							

Dai YN 2019 [28]	T:44	T:30–67	NR	II∼III	TC1A	CT + CKI 20 mL, qd,6d	8 cycles	Unclear	ORR, DCR, HRQoL-KPS, ADRs, CEA/CA153 level
	C:44	C:30–67							

Dai YN 2020 [29]	T:50	T:31–67	NR	II∼III	TC1A	CT + CKI 20 mL, qd,6d	8 cycles	Unclear	ORR, DCR
	C:50	C:30–66							

Huang YH 2012 [30]	T:20	T:33–75	NR	III∼IV	TA	CT + CKI 20 mL, qd,14d	2 cycles	WHO	ORR, DCR, HRQoL-KPS, ADRs
	C:20	C:33–75							

Li LY 2016 [31]	T:34	T:45.6 ± 4.2	T:5.2 ± 2.1 y	III∼IV	TA	CT + CKI 20 mL, qd,14d	2 cycles	WHO	ORR, DCR, HRQoL-KPS, ADRs
	C:34	C:46.5 ± 3.1	C:6.2 ± 3.1 y						

Li YX 2012 [32]	T:30	T:49.3 ± 0.9	NR	II∼IV	C1EF	CT + CKI 15 mL, qd,14d	2 cycles	WHO	ORR, DCR, HRQoL-KPS, ADRs
	C:30	C:49.3 ± 0.9							

Ma YQ 2011 [33]	T:32	T:28–63	T:10m	III∼IV	TC1A	CT + CKI 20 mL, qd,21d	3 cycles	Unclear	HRQoL-KPS, ADRs
	C:31	C:30–62	C:11m						

Mao WJ 2018 [34]	T:39	T:40–70	NR	NR	TC1A	CT + CKI 15 mL, qd,12d	8 cycles	RECIST	ORR, DCR, ADRs, CEA/CA153 level
	C:39	C:40–70							

Niu YL 2017 [35]	T:40	T:50.02 ± 12.11	NR	NR	GP	CT + CKI 20 mL, qd,14d	3 cycles	Unclear	HRQoL-KPS
	C:40	C:48.35 ± 12.23							

Qi JH 2012 [36]	T:57	T:35–75	NR	IV	TP	CT + CKI 20 mL, qd,14d	2 cycles	WHO	ORR, DCR, HRQoL-KPS, ADRs
	C:52	C:35–75							

Qian XY 2017 [37]	T:23	T:46.6 ± 5.2	NR	IV	GP	CT + CKI 20 mL, qd,10d	2 cycles	RECIST	ORR, DCR, HRQoL-KPS, ADRs
	C:23	C:47.3 ± 5.9							

Ren JH 2010 [38]	T:62	T:39–65	NR	II∼III	C1AF	CT + CKI 30 mL, qd,15d	6 cycles	Unclear	HRQoL-KPS, ADRs, CEA/CA153 level
	C:60	C:39–65							

Ren MY 2016 [39]	T:50	T:50.34 ± 5.13	NR	IV	TE	CT + CKI 20 mL, qd,10d	2 cycles	WHO	ORR, DCR, ADRs
	C:50	C:50.19 ± 5.24							

Song RF 2009 [40]	T:64	T:22–65	NR	IV	NG	CT + CKI 20 mL, qd,20d	2 cycles	WHO	ORR, DCR, HRQoL-KPS
	C:54	C:24–63							

Sun X 2008 [41]	T:38	T:28–69	NR	IV	TA	CT + CKI 20 mL, qd,10d	2 cycles	Unclear	ORR, DCR, HRQoL-KPS, ADRs
	C:32	C:28–69							

Sun XH 2019 [42]	T:41	T:49.05 ± 7.29	NR	II∼III	TEC1	CT + CKI 20 mL, qd,7d	3 cycles	WHO	ORR, DCR, ADRs, CEA/CA153 level
	C:39	C:49.69 ± 6.11							
Wang J 2019 [43]	T:47	T:47.2 ± 5.3	NR	II∼III	TE	CT + CKI 20 mL, qd,10d	2 cycles	UICC	ORR, DCR, HRQoL-BREF, PFS, OS, ADRs, CEA/CA153 level
	C:47	C:45.3 ± 4.9							

Wang L 2007 [44]	T:30	T:28–65	NR	NR	C1EF	CT + CKI 20 mL, qd,10d	2–3 cycles	WHO	ORR, DCR, ADRs
	C:30	C:28–65							

Wei YH 2010 [45]	T:12	T:35–65	NR	NR	C1AF	CT + CKI 20 mL, qd,15d	2 cycles	Unclear	HRQoL-KPS, ADRs
	C:12	C:35–65							

Xu N 2017 [46]	T:52	T:42.4 ± 4.5	NR	II∼III	C1AF	CT + CKI 20 mL, qd,21d	6 cycles	RECIST	ORR, DCR, ADRs
	C:52	C:43.7 ± 5.3							

Xu HJ 2017 [47]	T:49	T:28–75	NR	II∼III	TC1A	CT + CKI 12 mL, qd,10d	4 cycles	Unclear	HRQoL-KPS, ADRs
	C:49	C:26–76							

Yan J 2015 [48]	T:40	T:52.3 ± 4.6	NR	II∼III	TE	CT + CKI 12 mL, qd,14d	2 cycles	WHO	ORR, DCR, HRQoL-KPS, ADRs
	C:40	C:52.5 ± 4.5							

Yang X 2013 [49]	T:30	T:41.5 ± 10.29	NR	III∼IV	TE	CT + CKI 20 mL, qd,21d	NR	RECIST	ORR, DCR, HRQoL-KPS, ADRs
	C:30	C:41.5 ± 10.29							

Yang H 2019 [50]	T:55	T:44.1 ± 4.5	NR	II∼III	TA	CT + CKI 20 mL, qd,7d	6 cycles	WHO	ORR, DCR, ADRs
	C:55	C:43.5 ± 4.7							

Zhai XJ 2014 [51]	T:61	T:42.7 ± 10.5	NR	I∼III	C1AF	CT + CKI 20 mL, qd,21d	6 cycles	Unclear	ADRs
	C:62	C:43.5 ± 11.2							

Zhang ZJ 2015 [52]	T:65	T:43.2 ± 17.9	NR	I∼II	AC1	CT + CKI 12 mL,21d	6 cycles	Unclear	HRQoL-KPS, ADRs
	C:65	C:43.2 ± 17.9							

Zhang GY 2014 [53]	T:36	T:46.25 ± 5.29	T:8.25 ± 2.28	NR	TC1A	CT + CKI 30 mL, qd,14d	6 cycles	RECIST	ORR, DCR, HRQoL-KPS, ADRs
	C:36	C:46.36 ± 5.41	C:8.16 ± 2.3						

Zhang JZN 2018 [54]	T:45	T:34–72	NR	IV	GC2	CT + CKI 20 mL, qd,10d	4 cycles	RECIST	ORR, DCR, HRQoL-KPS, ADRs, CEA/CA153 level
	C:45	C:33–71							

*Note.* T: compound KuShen injection group; C: control group; d: day; y: year; NR: not reported. ∗: chemotherapy regimens: A: ADM (doxorubicin); F: 5-fluorouracil; C1: CTX (cyclophasphamide); T: docetaxel; E: EPI (epirupicin); G: GEM (gemcitabline); P: paclitaxel; N: NVB (vinorelbine); C2: CBP (carboplatin). HRQoL-KPS: health-related quality of life measured by Karnofsky Performance scale; HRQoL-BREF: health-related quality of life measured by QoL-BREF scale.

**Table 2:** Effect estimates of compound Kushen injection for breast cancer.

Outcomes and comparisons		Studies	Participants	Effect estimate (95%CI) REM	*P* value	Study ID references
*Compound Kushen injection plus chemotherapy versus chemotherapy alone*						
ORR	Stage II∼III	7	656	RR 1.31, [1.17, 1.46], *I*^*2*^ = 0%	*P*=0.002	[28, 29, 42, 43, 46, 48, 50]
	Stage IV	6	533	RR 1.33, [1.11, 1.60], *I*^*2*^ = 0%	*P*=0.002	[36, 37, 39–41, 54]

DCR	Stage II∼III	7	656	RR 1.20, [1.12, 1.28], *I*^*2*^ = 0%	*P* < 0.001	[28, 29, 42, 43, 46, 48, 50]
	Stage IV	6	533	RR 1.22, [1.11, 1.35], *I*^*2*^ = 0%	*P* < 0.001	[36, 37, 39–41, 54]

ORR	WHO criteria	10	825	RR 1.36, [1.18, 1.56], *I*^*2*^ = 2%	*P* < 0.001	[30–32, 36, 39, 40, 42, 44, 48, 50]
	RECIST criteria	6	450	RR 1.40, [1.16, 1.68], *I*^*2*^ = 0%	*P* < 0.001	[34, 37, 46, 49, 53, 54]

DCR	WHO criteria	10	825	RR 1.17, [1.08, 1.28], *I*^*2*^ = 30%	*P* < 0.001	[30–32, 36, 39, 40, 42, 44, 48, 50]
	RECIST criteria	6	450	RR 1.35, [1.21, 1.52], *I*^*2*^ = 0%	*P* < 0.001	[34, 37, 46, 49, 53, 54]

KPS score improvement rate	Stage II∼III	5	468	RR 2.93, [1.88, 4.56], *I*^*2*^ = 0%	*P* < 0.001	[28, 38, 42, 47, 48]
	Stage IV	4	343	RR 2.98, [1.88, 4.72], *I*^*2*^ = 0%	*P* < 0.001	[36, 37, 40, 41]

QoL-BREF score		1	94	MD 18.11, [16.50, 19.72]	*P* < 0.001	[43]
PFS		1	94	MD 2.24, [1.26, 3.22]	*P* < 0.001	[43]
OS		1	94	MD 2.24, [1.45, 3.43]	*P* < 0.001	[43]
ADRs	Leukocyte decrease	15	1121	RR 0.60, [0.5, 0.71], *I*^*2*^ = 40%	*P* < 0.001	[27, 30–34, 36, 37, 39, 41, 42, 47, 48, 53, 54]
	Platelet decrease	9	750	RR 0.41, [0.29, 0.58], *I*^*2*^ = 36%	*P* < 0.001	[27, 28, 36, 37, 39, 42, 47, 53, 54]
	Liver injury	13	1215	RR 0.42, [0.31, 0.57], *I*^*2*^ = 26%	*P* < 0.001	[26–28, 36, 37, 39, 42–44, 46, 50–52]
	Renal injury	9	879	RR 0.63, [0.46, 0.86], *I*^*2*^ = 0%	*P*=0.004	[27, 28, 36, 37, 42, 46, 49–51]
	Nausea and vomiting	14	1171	RR 0.68, [0.59, 0.79], *I*^*2*^ = 43%	*P* < 0.001	[26, 33, 34, 36, 37, 41, 43, 44, 46, 47, 49, 51, 53, 54]
	Diarrhea	3	210	RR 0.55, [0.35, 0.88], *I*^*2*^ = 0%	*P* < 0.001	[26, 37, 49]
	Alopecia	8	584	RR 0.51, [0.39, 0.67], *I*^*2*^ = 32%	*P* < 0.001	[27, 28, 33, 34, 36, 37, 49, 50, 53]
	Oral mucositis	4	441	RR 0.18, [0.07, 045], *I*^*2*^ = 0%	*P* < 0.001	[26, 46, 50, 51]
Tumor marker	Decrease rate of CEA	3	336	RR 1.19, [1.05, 1.35], *I*^*2*^ = 0%	*P*=0.007	[25, 28, 52]
	Decrease rate of CA153	2	206	RR 1.15, [1.03, 1.27], *I*^*2*^ = 0%	*P*=0.009	[25, 29]

Note: REM: random-effect models; CI: confidence intervals; MD: mean difference; RR: risk rate.

**Table 3:** Summary of main findings of RCTs on compound Kushen injection for breast cancer.

Outcomes	No. of participants (no. of RCTS)	Certainty of the evidence	Relative effect (95% CI)	Anticipated absolute effects	
				Risk with control	Risk difference with intervention (95% CI)
*CKI plus chemotherapy versus chemotherapy*					
ORR	1694 (21)	⊕⊕○○^1,5^	RR 1.30, [1.18, 1.43]	471 per 1000	141 more per 1000, (from 85 more to 203 more)
DCR	1627 (20)	⊕○○○^1,2,5^	RR 1.21, [1.15, 1.28]	698 per 1000	147 more per 1000, (from 105 more to 196 more)
KPS score improvement rate	1172 (14)	⊕⊕○○^1,5^	RR 1.42, [1.26, 1.61]	476 per 1000	200 more per 1000, (from 124 more to 291 more)
KPS score	393 (6)	⊕○○○^1,4,5^	N/A	N/A	The KPS score improvement in the intervention groups was 12.81 higher (10.16 to 15.46 higher)
PFS	94 (1)	⊕○○○^1,4,5^	N/A	N/A	The PFS in the intervention groups was 2.24 months higher (1.26 to 3.22 higher)
OS	94 (1)	⊕○○○^1,4,5^	N/A	N/A	The OS in the intervention groups was 2.44 months higher (1.45 to 3.43 higher)
Leukocyte decrease	1121 (15)	⊕○○○^1,2,5^	RR 0.60, [0.5, 0.71]	572 per 1000	229 fewer per 1000, (from 286 fewer to 166 fewer)
Platelet decrease	750 (9)	⊕⊕○○^1,5^	RR 0.41, [0.29, 0.58]	397 per 1000	234 fewer per 1000, (from 282 fewer to 167 fewer)
Liver injury	1215 (13)	⊕⊕○○^1,5^	RR 0.42, [0.31, 0.57]	269 per 1000	156 fewer per 1000, (from 185 fewer to 116 fewer)
Renal injury	879 (9)	⊕⊕○○^1,5^	RR 0.63, [0.46, 0.86]	203 per 1000	83 fewer per 1000, (from 116 fewer to 37 fewer)
Nausea and vomiting	1171 (14)	⊕⊕○○^1,5^	RR 0.68, [0.59, 0.79]	578 per 1000	185 fewer per 1000, (from 237 fewer to 121 fewer)
Diarrhea	210 (3)	⊕○○○^1,3,5^	RR 0.55, [0.35, 0.88]	324 per 1000	146 fewer per 1000, (from 210 fewer to 39 fewer)
Alopecia	584 (8)	⊕○○○^1,2,5^	RR 0.51, [0.39, 0.67]	514 per 1000	252 fewer per 1000, (from 313 fewer to 170 fewer)
Oral mucositis	441 (4)	⊕⊕○○^1,5^	RR 0.18, [0.07, 045]	136 per 1000	111 fewer per 1000, (from 126 fewer to 75 fewer)
Decrease rate of CEA	336 (3)	⊕○○○^1,3,5^	RR 1.19, [1.05, 1.35]	468 per 1000	89 more per 1000, (from 23 more to 164 more)
Decrease rate of CA153	206 (2)	⊕○○○^1,3,5^	RR 1.15, [1.03, 1.27]	821 per 1000	123 more per 1000, (from 25 more to 22 more)

*Note.* (1) Risk of bias: methodological quality of these trials was graded as “high risk of bias” due to the design of comparison. The trials also had unclear risk of performance bias for not reporting blinding the outcome assessor. (2) Inconsistency: the signiﬁcant heterogeneity with a large I2 value, an I2 >50% indicated the possibility of statistical heterogeneity among the studies. (3) Imprecision: for dichotomous outcomes, the total number of events is less than 300; or pooled results included no effects. (4) Imprecision: for continuous outcomes, the total population size is less than 400; or pooled results included no effects. (5) All the trials had high risk of performance bias for not blinding the participants. ^*∗*^The risk in the intervention group (and its 95% confidence interval) is based on the assumed risk in the comparison group and the relative effect of the intervention (and its 95% CI). CI: confidence interval; RR: risk ratio; MD: mean difference. N/A: not applicable. RCT: randomized controlled trial. No.: number. ⨁: very low quality of the evidence; ⨁⨁: low quality of the evidence; GRADE Working Group grades of evidence. Low quality: our confidence in the effect estimate is limited: the true effect may be substantially different from the estimate of the effect. Very low quality: we have very little confidence in the effect estimate; the true effect is likely to be substantially different from the estimate of effect.

## Data Availability

The data used to support the findings of this study are available from the corresponding author upon request.

## Abbreviations

ADRs: Adverse drug reactions
CKI: Compound Kushen injection
CONSORT: Consolidated Standards of Reporting Trials
CI: Confidence interval
CNKI: China National Knowledge Infrastructure
DCR: Disease control rate
HRQoL: Health-related quality of life
KPS: Karnofsky Performance Scale
MD: Mean difference
ORR: Objective response rate
OS: Overall survival
PFS: Progression-free survival
RCTs: Randomized clinical trials
RR: Risk ratio
REM: The random-effect model
SinoMed: Chinese Biomedical Literature Database
SE: Standard error
TSA: Trial sequential analysis
TCM: Traditional Chinese medicine
VIP: China Science Technology Journal Database.
